# PM_2.5_ and Kidney Function: Long-Term Exposures May Lead to Modest Declines

**DOI:** 10.1289/ehp.124-A168

**Published:** 2016-09-01

**Authors:** Nate Seltenrich

**Affiliations:** Nate Seltenrich covers science and the environment from Petaluma, CA. His work has appeared in *High Country News*, *Sierra*, *Yale Environment 360*, *Earth Island Journal*, and other regional and national publications.

Exposure to fine particulate matter (PM_2.5_) is associated with cardiovascular health impacts including increased risk of irregular heartbeat and pulmonary embolism (arterial blockage).^1^ However, the relationship between PM_2.5_ and renal function, an independent cardiovascular risk factor^23^ and significant health metric in its own right,^4^ ^,^ ^5^ ^,^ ^6^ ^,^ ^7^ is poorly understood. A new longitudinal study offers early evidence that PM_2.5_ exposure is associated with lower kidney function and a higher rate of kidney function decline over time.^8^

“While there is evidence that the association between ambient particulate matter and cardiovascular disease may be explained by several pathways at the molecular or functional level, the underlying mechanisms that may explain the association remain to be fully elucidated,” says lead author Amar Mehta, a visiting scientist at the Harvard T.H. Chan School of Public Health.

**Figure:**
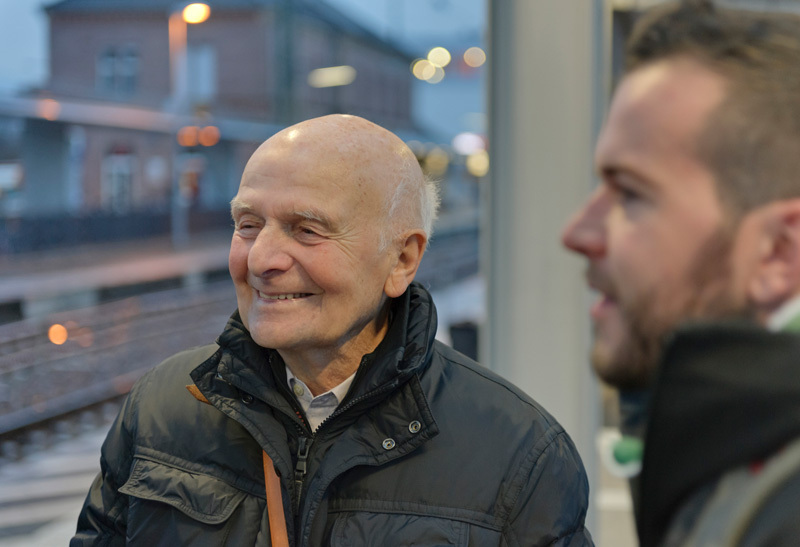
Reductions in renal function like those seen in the older men in this study probably would not harm individuals with healthy kidneys. However, they could set the stage for cardiovascular impacts in the elderly. © Albrecht Weißer/Getty Images

The research team compared PM_2.5_ exposure and renal function for 669 predominately white men with a mean age of 73.5 years enrolled in the Boston-based Department of Veterans Affairs Normative Aging Study.^9^ At up to 4 physical examinations between 2000 and 2011, each participant had his serum creatinine levels read and eGFR (estimated glomerular filtration rate) calculated as a measure of renal function. The researchers estimated participants’ exposure to PM_2.5_ over the year prior to each visit, based on the men’s home addresses and incorporating high-resolution satellite data.

Participants’ median 1-year PM_2.5_ exposure levels ranged from approximately 7.5 to 12.5 µg/m^3^. By comparison, the primary National Ambient Air Quality Standard for 1 year is 12 µg/m^3^ averaged over 3 years.^10^ This standard is designed to be protective of all groups of people, including the elderly. For this particular population, the authors estimated that a 2.1-µg/m^3^ increase in PM_2.5_ over a 1-year period was associated with a reduction in eGFR comparable to that seen with a 2-year increase in age in the same men.^8^

Joel Kaufman, a physician, epidemiologist, and professor at the University of Washington, questions the clinical significance of the change in eGFR as far as renal function goes. Based on the evidence so far, he says, “these air pollution levels are not going to cause someone with normal kidney function to need dialysis.” Kaufman was not involved in the study.

On the other hand, the implications for cardiovascular disease risk could potentially be significant. Brown University associate professor Gregory Wellenius, who coauthored a 2013 paper examining associations between air pollution and renal function,^11^ says, “The effect is small numerically, but when you compare it to a two-year aging of an individual, it isn’t trivial in this elderly and potentially vulnerable population. And when you apply that over an entire population, the effect can be substantial.”

These issues leave the door open for further research. Future work could seek to replicate the findings in other cohorts, or explore the same issue in a similar cohort experiencing higher exposures, with an eye toward identifying clinically relevant impacts on the kidney—plus connecting the dots between air pollution, renal function, and cardiovascular disease. “I think it’s interesting to look at kidney disease and environmental exposures in general,” Kaufman says, “because kidney disease is becoming an important source of morbidity and mortality as our population ages.”
